# Exploitative and Hierarchical Antagonism in a Cooperative Bacterium

**DOI:** 10.1371/journal.pbio.0030370

**Published:** 2005-11-01

**Authors:** Francesca Fiegna, Gregory J Velicer

**Affiliations:** **1**Max-Planck Institute for Developmental Biology, Tüebingen, Germany; Princeton UniversityUnited States of America

## Abstract

Social organisms that cooperate with some members of their own species, such as close relatives, may fail to cooperate with other genotypes of the same species. Such noncooperation may take the form of outright antagonism or social exploitation. *Myxococcus xanthus* is a highly social prokaryote that cooperatively develops into spore-bearing, multicellular fruiting bodies in response to starvation. Here we have characterized the nature of social interactions among nine developmentally proficient strains of *M. xanthus* isolated from spatially distant locations. Strains were competed against one another in all possible pairwise combinations during starvation-induced development. In most pairings, at least one competitor exhibited strong antagonism toward its partner and a majority of mixes showed bidirectional antagonism that decreased total spore production, even to the point of driving whole populations to extinction. Differential response to mixing was the primary determinant of competitive superiority rather than the sporulation efficiencies of unmixed populations. In some competitive pairings, the dominant partner sporulated more efficiently in mixed populations than in clonal isolation. This finding represents a novel form of exploitation in bacteria carried out by socially competent genotypes and is the first documentation of social exploitation among natural bacterial isolates. Patterns of antagonistic superiority among these strains form a highly linear dominance hierarchy. At least some competition pairs construct chimeric, rather than segregated, fruiting bodies. The cooperative prokaryote *M. xanthus* has diverged into a large number of distinct social types that cooperate with clone-mates but exhibit intense antagonism toward distinct social types of the same species. Most lengthy migration events in nature may thus result in strong antagonism between migratory and resident populations, and this antagonism may have large effects on local population sizes and dynamics. Intense mutual antagonism appears to be more prevalent in this prokaryotic social species than has been observed in the eukaryotic social slime mold *Dictyostelium discoideum,* which also exhibits multicellular development. The finding of several cases of facultative social exploitation among these natural isolates suggests that such exploitation may occur frequently in nature in many prokaryotes with cooperative traits.

## Abstract 2

Experimentally competing different natural strains of the social bacterium *Myxococcus xanthus* reveals that some strains exploit others, with implications for the evolution of intraspecific cooperation and the generation of bacterial diversity.

## Introduction

The microbial world is replete with cooperative behaviors that appear to produce density-dependent fitness benefits [1], including biofilm formation [2], quorum sensing [3], siderophore production [4,5], and fruiting body construction [6–8]. Also present, however, are strong negative social interactions that have evolved repeatedly among distinct lineages of relatively asocial species of bacteria such as *Escherichia coli* [9]. In highly social bacteria, such as those that form multicellular fruiting structures*,* the degree of social compatibility or antagonism among divergent strains classified within a single species remains unexplored. In the eukaryotic social slime mold *Dictyostelium discoideum,* which produces spores within multicellular fruiting bodies upon starvation, total social productivity (i.e., spore production) does not appear to suffer when distinct genotypes are mixed [10,11]. It is unclear whether intraspecific mixing of multicellular prokaryotes is similarly benign with respect to the benefits of social development or rather has more severe effects at the population level. It is also unknown whether natural, socially competent genotypes of a cooperative bacterial species are capable of exploiting other genotypes of the same species in a social context.

The soil-dwelling myxobacteria are unique among prokaryotes in the variety and sophistication of their social behaviors. They swarm cooperatively throughout a vast range of global soil ecosystems [12] while feeding on other microorganisms and detritus [13]. Upon starvation, local groups aggregate and develop into multicellular fruiting bodies [6]. In the model species *Myxococcus xanthus,* stress-resistant spores are formed by a minority of the fruiting body population, whereas the remainder appear to either undergo suicidal autolysis or remain undifferentiated [14].

The motility of *M. xanthus* and external migration vectors such as animals, insects, water, and wind should frequently cause distinct genotypes to encounter one another over both small and large spatial scales. Such encounters may result in a wide variety of interactions, including neutral social compatibility, antagonism, synergism, and social exploitation of one genotype by another. Smith and Dworkin [15] previously showed that two distinct *Myxococcus* species *(M. xanthus* and*M. virescens)* are incompatible during cooperative development. Clones of these two species separate into distinct, unmixed fruiting bodies in initially mixed cultures, and *M. virescens* strongly dominates over *M. xanthus* in spore production. The dominance of *M. virescens* is largely due to the production of compounds toxic to *M. xanthus*. The nature of social interactions among isolates classified within a single species of myxobacteria, however, remains unclear.

In this study, we quantify the effect of pairwise mixing among nine developmentally proficient global isolates of *M. xanthus* on the developmental performance of these strains and address the following questions. (i) Do most strains respond positively, negatively, or neutrally to mixing with other genotypes? (ii) Do competitors segregate into distinct fruiting bodies or mix within individual fruiting bodies? (iii) In any given pair, do both strains respond similarly to mixing (e.g., negatively), or are there cases where one competitor exploits another by simultaneously showing enhanced performance in mixture while inhibiting the performance of the other? (iv) Which better predicts the outcome of mixed developmental competitions: the relative performance of two competitors in clonal isolation or the relative effect of mixing on two competitors' developmental performance? (v) What effects do observed antagonisms have on the total social productivity (i.e., spore production) of competitive mixtures relative to clonal controls? (vi) Are most fitness relationships among three or more strains transitive (i.e., linear) or nontransitive (i.e., circular)?

Paired competitors were mixed at equal proportions in all possible combinations at the onset of starvation-induced development. Previous theory has predicted that antagonism among bacteriocin-producing competitors should be highest when the degree of relatedness within a mixed group is approximately 0.5 [16], suggesting that equal proportions of pairwise competitors may be the optimal condition for detecting any antagonism that exists between them. Mixing effects on fruiting body formation were observed, and the sporulation efficiency of each strain in mixture was contrasted to its spore production in isolation. The majority of strain interactions were strongly negative, and many pairings resulted in bidirectional inhibition of sporulation. In some pairings, one competitor performed better in mixed competition than in clonal isolation, revealing the capacity for social exploitation among natural prokaryotes and demonstrating that socially competent bacteria are capable of exploiting other genotypes. All except one set of three-way fitness relationships were linear rather than circular, supporting the view that the social incompatibilities among these genotypes are the result of local adaptation by isolated lineages.

## Results

### Mixing Effects on Population Morphology

Strong strain-by-strain interactions were clearly evident in the effects of mixing on the shape, size, and distribution of fruiting bodies in mixed cultures relative to their corresponding pure cultures (Figure 1). In almost all competition pairs, the developmental morphology of the mixed population was clearly distinct from those of both pure-culture counterparts. Mixed populations usually showed fewer fruiting bodies than each competitor in isolation, thus illustrating that cooperative development is normally less efficient when performed by chimeric, rather than clonal, populations. Because sporulation efficiency decreases at lower cell densities, we tested whether the effects of mutual antagonism are even more severe when mixed populations begin development at reduced density (10^9^ cells/ml). Under these conditions, mixing of strains E and F completely eliminated developmental aggregation (Figure 2).

**Figure 1:**
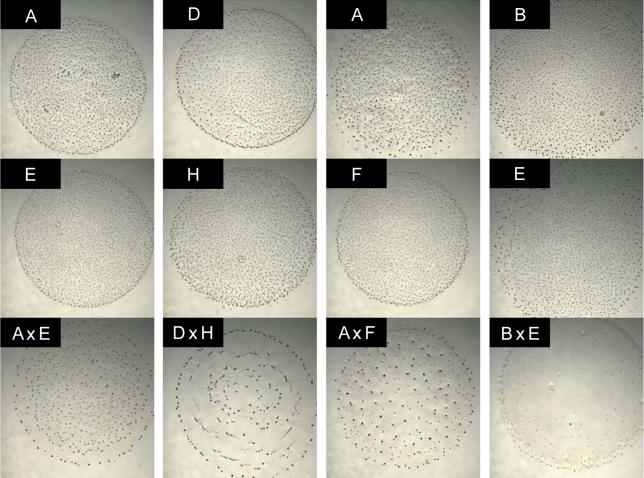
Effects of Pairwise Mixing on Fruiting Body Size and Distribution Pairings AE, DH, AF, and BE are shown. Pure-culture fruiting bodies are shown in the top and middle photographs of each column (first and second listed strains, respectively), and fruiting bodies of the corresponding mixed culture are shown at the bottom.

**Figure 2:**
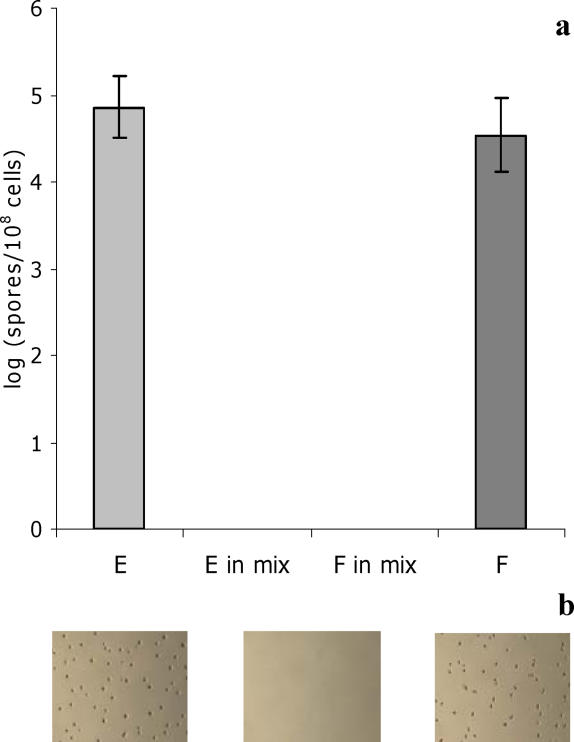
Extinction Caused by Mutual Antagonism between Strains E and F (A) At an initial developmental density of 10^9^ cells/ml, no spores survived starvation in mixed populations, but both competitors performed well in clonal isolation. Error bars indicate 95% confidence intervals. (B) Developmental phenotypes of E and F in pure culture (left and right, respectively) and in mixed competition (middle).

### Sporulation Mixing Effects

The use of antibiotic resistance markers allowed us to measure the effects of mixing on both the total spore production of mixed populations and each competitor individually. Mixing decreased the overall social productivity (i.e., total spore production) of mixed populations (Table 1). Across the 36 competition pairs, the average total spore production by mixed populations was lower (57.2%) than that expected from spore production in the pure-culture controls (*p* = 0.0002, two-tailed one-sample *t*-test of nine strain averages, *df* = 8). Four pairs sporulated at less than 10% of expected levels (1.6%-6.8%), 14 pairs produced between 10% and 50% of the expected spore number, and 16 pairs produced between 53% and 87%. One pairing (EF) was repeated at a lower initial density of 10^9^ cells/ml. No spores of either competitor survived development, whereas both competitors produced many spores in pure culture at the same density (Figure 2). At the standard higher density of 5 × 10^9^ cells/ml, two pairings appeared to have enhanced spore production, but neither increase was statistically significant.

**Table 1:**
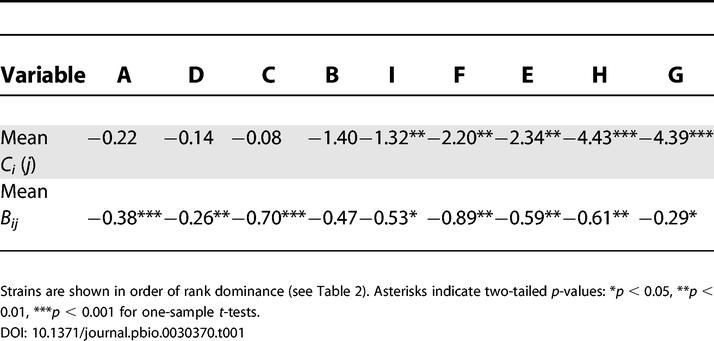
Average One-Way (*C_i_* [*j*]) and Bidirectional *(B_ij_)* Mixing Effects by Strain

Of 72 one-way mixing effects (*C_i_*[*j*]), 56 were negative (strains sporulated less efficiently in mixture than in isolation), whereas 16 were positive (strains sporulated more efficiently in mixture than in isolation) (Figure 3). Thus, 20 pairs exhibited bidirectional antagonism, and 16 pairs had one positive response and the other negative (strain pairs AB, AF, AG, AI, BG, BI, CF, CG, CI, DB, DF, DG, DH, DI, EG, IG [positive response strain listed first in each pair]). The mean one-way mixing effect of each strain was negative for all nine strains (Table 1), and the grand mean of one-way mixing effects was strongly negative (−1.85, *p* = 0.0010, two-tailed one-sample *t*-test of nine strain averages, *df* = 8) and corresponded to an approximately 70-fold average decrease in sporulation efficiency in mixed competitions relative to pure-culture development.

**Figure 3:**
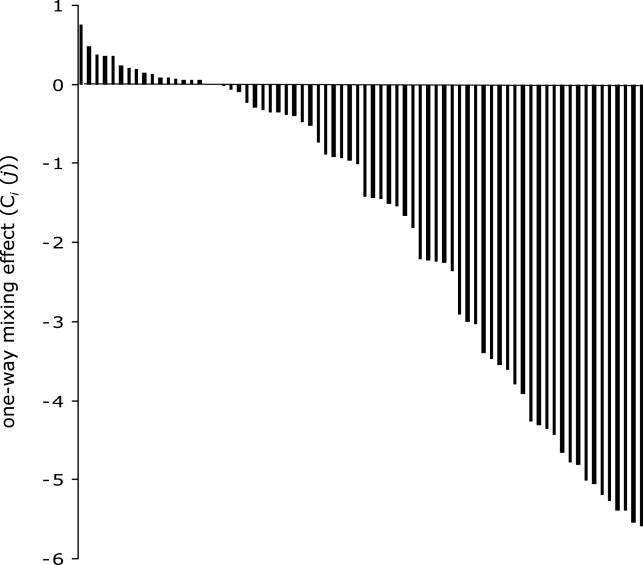
Distribution of Mixing Effects on the Sporulation Efficiencies of Individual Competitors (C*_i_*[*j*])

The 16 positive values corresponded to competition mixes in which one strain appeared to sporulate more efficiently in mixture with a competitor than in clonal isolation and simultaneously inhibited its competitor's performance. These outcomes provide evidence of facultative social exploitation in bacteria. By “facultative,” we mean that the ability to exploit other genotypes is not derived from a genetic social defect that prevents normal cooperation under clonal conditions. Thus, the exploiters do not obligately defect from cooperation in all contexts but do so only in mixes with some genetically distinct social partners.

The distributions of strain representations among the 16 exploitative responses and the 16 corresponding negative responses by the inferior (exploited) partners are nonrandom, with some strains being disproportionately represented (*p* < 0.0001 in both cases, chi-square test). Among the 16 positive responses, 12 are made by strains A, C, and D (four, three, and five cases, respectively). These are the three most dominant competitors among the nine strains examined here (see explanation of Table 2 below). Inversely, among the partners exploited by dominant strains, strain G accounts disproportionately for six of the 16 apparent cases of exploitation.

**Table 2:**
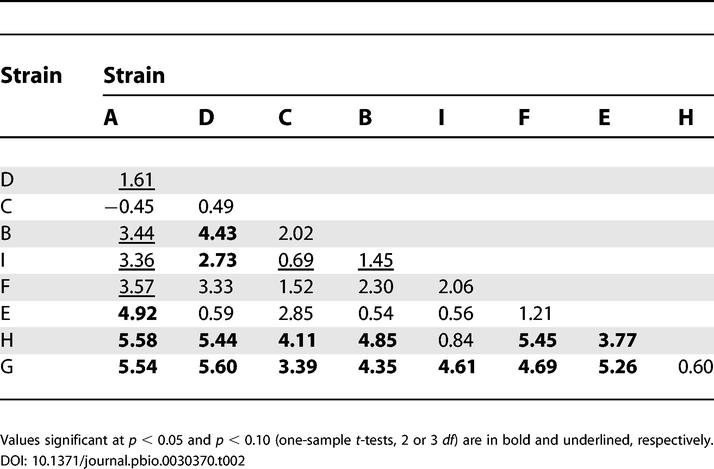
Relative Sporulation Efficiency *(W_ij_)* Dominance Hierarchy

Exploitative responses to mixing were demonstrated more robustly in two particular strain pairs that involved relatively strong positive responses in the larger matrix of competition mixes. Two competition pairings (DI and IG) with one strain showing a positive response to mixing were repeated with the dominant strain (D and I, respectively) at a lower initial frequency of 0.32 (Figure 4). Both D (vs. I) and I (vs. G) showed significantly increased sporulation efficiency in mixed competition relative to their efficiencies in pure culture (1.6- and 2.1-fold increases in efficiency, respectively; *p* = 0.04 and 0.005, respectively, one-tailed *t-*tests, *df* = 2).

**Figure 4:**
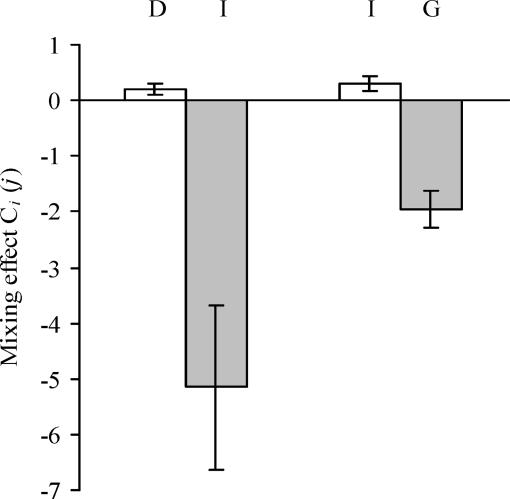
Facultative, Antagonistic Exploitation by Two Genotypes (D against I and I against G) during Mixed Development The log-scale effect of mixing strains *i* and *j* on the sporulation efficiency of strain *i* is given as *C_i_*(*j*). Open bars show the effect of mixing on sporulation efficiency for the dominant, exploitative competitor in each pair (D and I, respectively) in response to its inferior competitor. Shaded bars indicate the effect of mixing on the inferior strain (I and G, respectively). Error bars indicate 95% confidence intervals.

Among the 36 competition pairs initiated at a 50:50 ratio, very different mixing effect patterns were observed among all competitors against a given strain. Three examples are shown in Figure 5. Strain A showed dramatic inhibition of competitor sporulation in most pairings (greater than 1,000-fold mean inhibitory effect) but its own sporulation efficiency was relatively unaffected by these strains (Figure 5A). Strain B, in contrast, hindered other strains to approximately the same degree, on average, as its sporulation was hindered by the other strains (Figure 5B). Its interaction patterns, however, varied greatly among partners. Three competitors (A, C, and D) strongly inhibited the sporulation of B but were not harmed by the presence of B in return, while three other competitors (F, G, and H) showed the opposite pattern. The developmental performance of strain G was strongly inhibited by every competitor, with none but strain H being hindered by G in return (Figure 5C).

**Figure 5:**
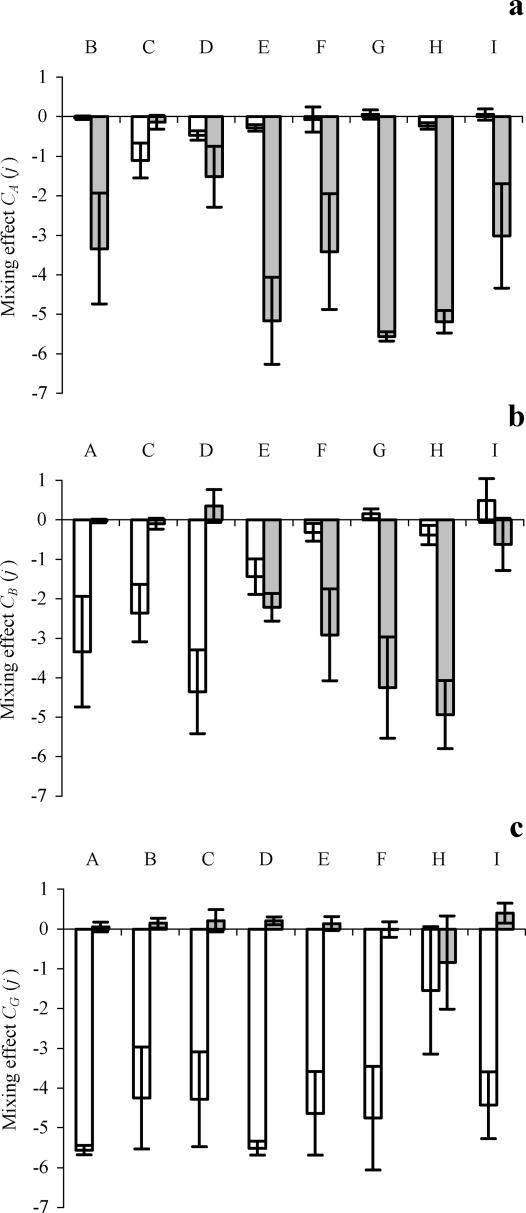
Bidirectional Mixing Effects for Strains A, B, and G against All Competitors The log-scale effect of mixing strains *i* and *j* on the sporulation efficiency of strain *i* is given as *C_i_*(*j*). Open bars show the effect of mixing on sporulation efficiency for strains A (A), B (B), and G (C) in response to each competitor shown along the horizontal axis. Shaded bars indicate the effect of mixing on the sporulation of the variable competitors. Error bars indicate 95% confidence intervals.

### Developmental Performance Predictors

We asked whether the relative performance of two competitors in clonal isolation or rather the relative effect of mixing better predicts the outcome of mixed developmental competitions. To compare the relative performance of the two strains within each competing pair, we calculated W*_ij_ (predicted relative sporulation), *W_ij_* (actual relative sporulation), and *C_ij_* (relative mixing effect) for each of the 36 unique competition pairings. From equations 1–6 (see Materials and Methods), *C_ij_* = *W_ij_* − W*_ij_. The relative effect of mixing on competing strains' sporulation efficiencies *(C_ij_)* is highly predictive of actual competitive performance *(W_ij_)* (*r* ^2^ = 0.967, *p <* 0.001, linear regression; Figure 6). In contrast, predicted relative performance based on pure-culture sporulation efficiencies (W*_ij_) showed no relationship with actual competitive performance *(W_ij_)* (*r* ^2^ = 0.010, *p =* 0.556, linear regression; Figure 6). These results show antagonistic social interaction between competing genotypes to be the primary determinant of actual relative sporulation efficiency.

**Figure 6:**
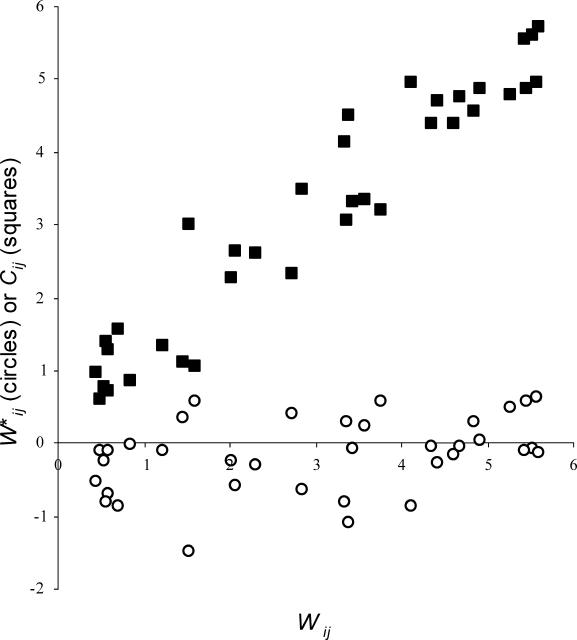
Relationships between Relative Mixing Effect *(C_ij_)* or Predicted Relative Performance (W*_ij_) (*y*-Axis) and Actual Relative Performance (*W_ij_*) (*x*-Axis)

### Dominance Hierarchies

We examined whether the structure of competitive dominance relationships across all pairwise comparisons is strongly hierarchical (i.e., linear). For measures of relative mixing effect *(C_ij_)* and relative sporulation efficiency in mixture *(W_ij_),* each strain was ranked by its number of positive *C_ij_* or *W_ij_* values among competitions with the other eight strains. The two resulting matrices are identical in structure and reveal a clear dominance hierarchy among the strains. Table 2 shows the *W_ij_* values of the competitors listed by column *(i)* relative to the competitors listed by row *(j).* Positive *W_ij_* values indicate that the column strain is superior to the row strain, whereas the only negative value indicates that the column strain (A) is inferior to the row strain (C). Values not shown in the opposite half of the matrix are simply the negative of the value shown in the reciprocal cell. For example, *W_DB_* = 4.43 (shown), whereas *W_BD_* = −4.43 (not shown). Both values mean that strain D produces approximately 27,000-fold more spores than strain B in mixed development starting at a 50:50 ratio (log[27,000] = 4.43).

Strains A, D, and C shared the highest rank dominance (superiority over seven competitors each) and are ordered by decreasing average *W_ij_*. The remaining strains, B, I, F, E, H, and G, exhibit five, four, three, two, one, and zero cases of superiority, respectively. The *W_ij_* values among the three pairings of A, D, and C reflect the sole nontransitive triad (A beats D, D beats C, and C beats A) of performance relationships in each respective hierarchy. The top four competitors were all isolated from North America (as opposed to Eurasia), but such a ranking distribution has a 9.2% likelihood of occurring by chance (binomial test).

Kendall's technique [17] was used to test for significant linearity in the dominance hierarchy. This test is most commonly used to test for patterns of behavioral dominance in animals but has also been used to examine fitness among multiple isolates of *D. discoideum* [11]. The null hypothesis assumes that the relationship between any two particular strains does not predict anything about the relationship that each strain has with other strains. Statistical significance is derived from a chi-square test with *K* representing the degree of linearity on a scale from zero (total absence of linearity) to one (perfect linearity). With our group size of nine individuals, dominance hierarchies with 16 or fewer nontransitive triads exhibit significant linearity. Our hierarchy had only one such triad, representing extremely strong and significant linearity (*K* = 0.965, *p* < 0.001), meaning that this set of strains shows many distinct fitness ranks (seven of nine possible) rather than few.

### Competitor Distributions across Fruiting Bodies

Distinct antibiotic-resistance states of mixed competitors also allowed us to examine whether competitors ever mix within fruiting bodies or rather segregate during development after forced mixing in liquid. For two pairings (AC and BI) in which both competitors sporulated at relatively high levels in the mixed populations, we tested whether only one competitor could be found within individual fruiting bodies or rather both. For each pair*,* fruiting bodies were transferred to two nutrient agar plate types (ten to each type for AC and 40 to each type for BI), with each plate type containing distinct antibiotic corresponding to the competitors' resistance states. Fruiting bodies were then scored for subsequent growth after 5 d.

If competitors always segregate into separate fruiting bodies, only half of the total number of transferred fruiting bodies should germinate, regardless of the fruiting-body type ratio, because each fruiting body will germinate on only one of the two selective agar types. In the AC competition, all ten fruiting bodies germinated on whichever selective media they were placed, indicating that A and C form chimeric populations within fruiting bodies and likely do so in all fruiting bodies. The probability of all 20 fruiting bodies germinating under the total segregation hypothesis is extremely low (*p* < 0.0001, binomial test).

In the BI competition, however, the 40 fruiting bodies under selection for strain B (plates with rifampin) all germinated, whereas only three of the 40 fruiting bodies under selection for strain I (novobiocin plates) germinated. This result might reflect either total segregation of the competitors into distinct fruiting bodies (with I fruiting bodies in the minority) or segregation of B in most fruiting bodies with a small percentage of chimeras in which both B and I are present. Under the total segregation model, our novobiocin result gives the estimate that approximately 7.5% of fruiting bodies are of type I, which leads to the prediction that only 92.5% (37) of fruiting bodies will grow on rifampin agar. The total segregation hypothesis is unlikely to be correct, however, because the probability of all 40 fruiting bodies growing on rifampin when only 37 are expected is only 4.4% (binomial test). In the quantitative spore production assays, strain B sporulated approximately 28-fold more efficiently than strain I, averaged over all fruiting bodies and replicates. However, strain B was found in only 12.3-fold more fruiting bodies than strain I, suggesting that even if the total segregation hypothesis were correct, strain I should sporulate less efficiently within its respective fruiting bodies than strain B.

## Discussion

Intraspecific antagonism among relatively asocial bacteria is well known [9,18–20], but the nature of intraspecific interactions among divergent genotypes of a highly social bacterium has not been previously characterized. Here we have shown that intense antagonism occurs between distinct genotypes of the cooperative prokaryote *M. xanthus* under environmental conditions in which social cooperation is crucial to fitness and survival. This antagonism negatively affected social productivity in almost all cases, with several pairings of antagonistic competitors reducing total spore production by greater than 90%. Most individual responses to mixing were strongly negative, but some strains exploited others by performing better in mixture than in isolation. Overall fitness ranks among the isolates were strongly hierarchical, with the three best strains forming a dominant triad (with nontransitive fitness ranks within the triad) and the remaining six exhibiting a perfect hierarchy.

These patterns reveal that *M. xanthus* as a species is not composed of a small number of cooperative units. Rather, this species has diverged into a large number of distinct social types that cooperate with clone-mates and perhaps very close relatives but exhibit intense antagonism toward distinct social types of the same species. This leads to the prediction that most long-distance transports of soil particles will result in strong antagonism between migratory and resident populations of *M. xanthus*. Our results show that such antagonism often causes large reductions in the total spore production of chimeric populations and can even cause whole populations to become extinct (see Figure 2). In migratory encounters involving intense mutual antagonism, resulting reductions in total population size may jeopardize the prosperity or even survival of mixed populations when density-dependent cooperation is important for fitness, such as during starvation. Interestingly, chimeric mixing of distinct *D. discoideum* genotypes does not appear to reduce total spore production during its eukaryotic version of cooperative development, although group mobility is impaired by chimeric mixing [11]. This difference between *D. discoideum* and *M. xanthus* in the effects of mixing on spore production may reflect fundamental differences in the mechanisms of intraspecific competition between eukaryotic and prokaryotic social microorganisms more generally.

A previous *Myxococcus* study found that two genotypes classified as distinct species *(M. virescens* and *M. xanthus)* segregated into separate fruiting bodies after forced mixing and that the *M. virescens* isolate inhibited sporulation of the *M. xanthus* isolate [15]. In contrast, we have shown that at least some isolate pairs classified as *M. xanthus* are capable of forming chimeric fruiting bodies, suggesting that such chimerism might also occur under natural conditions, even if rarely. Such within-species chimerism has also been observed in *D. discoideum* fruiting bodies [7]. It has been suggested that spontaneous mixing might be advantageous for all involved genotypes under conditions in which fruiting is important for survival but in which distinct co-existing genotypes must combine in order to generate a group size larger than the minimum threshold required to undergo development [21]. Under such a scenario, even competitors that perform relatively poorly in mixed groups will be better off mixing than segregating, as they would produce at least some spores rather than none. When distinct genotype populations are all sufficiently large to sporulate efficiently as clonal groups, however, only dominant competitors would benefit from chimerism. The degree to which distinct *Myxococcus* genotypes may actually mix in the wild is under further investigation.

Until recently, myxobacterial classification was based exclusively on characterization of morphological traits, including the size, shape, and color of fruiting bodies and growing swarms. A recent comparison of a 16S rRNA-based phylogeny of the myxobacteria with traditional classification showed that the two approaches yielded largely consistent results across the monophyletic group [22]. At the genus level, however, the 16S rRNA approach did not always resolve strains bearing the same traditional species classification into a single group distinct from other strains with a different species classification. More comprehensive molecular-based approaches to classification are now feasible, including rapid whole-genome sequencing [23], and will allow a much finer resolution of species and strain distinctions within *Myxococcus* and other genera. Questions of future interest emerge from comparison of the *M. virescens-* vs.-*M. xanthus* study with our results, including whether genotypes of distinct species classifications ever form fruiting chimeras and whether qualitative antagonism patterns during interspecific competitions are consistent or highly variable across multiple isolates from each species.

Relatively asocial bacterial species such as *E. coli* have evolved intense forms of intraspecific antagonism [24]. Our results demonstrate that such antagonisms evolve readily in highly cooperative bacteria as well. In *E. coli,* the best known form of intraspecific antagonism is mediated by the production of colicin proteins from plasmid-borne genes that can be readily exchanged among genotypes via conjugative plasmid transfer [25]. In contrast, there is no evidence that *M. xanthus* hosts self-replicating plasmids [26], and *M. xanthus* has no known mechanism of conjugative DNA transfer between cells. Thus, both the evolutionary and biochemical mechanisms of antagonism are likely to be fundamentally different between these species.

At the biochemical level, *M. xanthus* carries genes for the production of a variety of secondary metabolites not present in most bacteria that may serve as anticompetitor agents [27]. Initial results indicate that the strains used in this study vary significantly in the types of secondary metabolites they produce (R. Müller and G. Velicer, unpublished data). This variation may be at least partially responsible for the asymmetric antagonisms observed here. Alternatively, inhibition of another competitor's sporulation may result from interference with the competitor's developmental signaling ability or motility rather than production of compounds that are directly toxic to competitor cells.

Regardless of its underlying biochemical mechanisms, the hierarchical antagonism observed during *M. xanthus* development among spatially isolated genotypes suggests that local diversity generated by migration of previously isolated genotypes will, on average, be short lived. Previous studies have shown that nontransitive fitness relationships among three or more competitors in structured habitats can serve to maintain biological diversity [18,20,28]. In our set of nine strains, however, only one triad of pairwise fitness relationships (among strains A, C, and D) of a total of 84 total triads showed nontransitivity. Competition experiments similar to those described here are under way with spatially proximate isolates of *M. xanthus,* and it will be of interest to determine whether nontransitivity is detected more frequently among close neighbors in the soil than was observed here among isolates from distant origins.

Although the average effect of competitive encounters here is strongly negative, 44% of competitive pairs had one partner that showed enhanced sporulation efficiency in response to mixing (see Figures 3 and 4). A variety of mechanisms might mediate such exploitation, including the ability to induce disproportionate cell death in a competitor and consume its remains as a growth substrate prior to spore differentiation. The exploitation observed here is facultative [29], whereas previous instances of bacterial cheating [4,8,30] have involved obligate defection from cooperation. Such obligate cheaters inherently (i.e., genetically) defect from cooperation regardless of the genotypic composition of their social neighbors (e.g., pure defectors or mixed defectors and cooperators). These obligate defectors perform poorly as clonal groups under selective conditions favoring sociality but perform well as a minority among cooperators. The isolates examined here, however, are highly proficient at sporulation in clonal isolation, but some are also able to exploit other genotypes in mixture.

Previous reports of bacterial social exploitation have involved genotypes modified or evolved in laboratory environments, whereas this study demonstrates intraspecific social exploitation among distinct natural bacterial isolates. The superiority of exploitative clones over their competitors in mixture observed here is not due to major differences in pure-culture performance. Rather, it is accomplished by the formidable competitive ability of some dominant genotypes to both inhibit (and in some cases, abolish) a competitor's sporulation ability and simultaneously convert a higher percentage of cells into spores specifically due to the presence of the inferior competitor.

## Materials and Methods

### Strains

Nine clonal isolates previously classified as *M. xanthus* based on morphological criteria (e.g., fruiting body color, shape, and size) were used in this work. DK801 (here, strain D, Tracy, California, United States), DK816 (B, Ontario, Canada), DK836 (A, Albany, New York, United States), and DK897 (E, Maryhill, Washington, United States) were obtained as clonal stocks from D. Kaiser; clones of Mxx15 (F, Olympia, Greece), Mxx23 (C, Minneapolis, Minnesota, United States), Mxx41 (G, Madras, India), Mxx104 (H, Bodrum, Turkey), and Mxx144 (I, Mt. Ar-Li, Taiwan) were purified from culture isolates provided by H. Reichenbach.

Three criteria were used in strain selection: (i) robust fruiting body formation and high sporulation efficiency, (ii) ability to obtain strain variants resistant to novobiocin, rifampin, or kanamycin, and (iii) zero or small effect of antibiotic resistance on sporulation efficiency. Strains were selected for use prior to collection of any data about their competitive ability against other strains. Strains A, B, D, E, and F were marked with spontaneous mutations conferring resistance to rifampin, while strains C, D, G, and I were likewise marked with spontaneous mutations to novobiocin resistance. Kanamycin resistance was conferred to strains C and E by chromosomal integration (via electroporation) of the plasmid pREG1727 [31] into the Mx8 phage attachment site and to strains D, F, and H by random chromosomal integration of the *mariner* transposon carried by the suicide plasmid pMycoMar [32]. Positions 46–445 of the 16S rRNA gene were sequenced in all nine strains and are identical to the standard *M. xanthus* lab strain DK1622.

In all reported results, estimates of marked strain performance in mixtures were compared to performance of the same marked strains in pure culture, not to pure cultures of unmarked parental strains. Nonetheless, it is worth noting that any marker effects on sporulation efficiency were small relative to total spore production (unpublished data). Factoring in marker effects to predict the outcome of hypothetical competitions between unmarked strains did not alter the structure of the *W_ij_* competition matrix (Table 2).

### Developmental competition assays

All agar plates were incubated at 32 °C and 90% rH, and all liquid cultures were grown at 32 °C, 300 rpm. Strains were grown on CTT agar medium [33] from ultralow freezer stocks, inoculated into CTT liquid, and kept in exponential-phase growth for 2 d. Cultures were then centrifuged at 20 °C for 15 min at 4,500 × g, and the pellets resuspended with TPM liquid [34] to a density of approximately 5 × 10^9^ cells/ml unless specified otherwise. For pure-culture assays, 100 μl of pure-culture resuspension was dispensed in the center of TPM plates (1.5% agar) for starvation. For paired competition assays, pure resuspended cultures from paired competitors were mixed at the appropriate ratio (50:50 in the complete matrix of pairwise mixes or 32:68 in a later experiment), and the total mixed volume (100 μl) was dispensed on the center of TPM plates. Thus, the total initial cell number and density were the same in competitive mixtures and clonal treatments. Developmental plates were incubated for 5 d, after which the cell populations were harvested with a scalpel blade, transferred into 1 ml of double-distilled H_2_O, and heated at 50 °C for 2 h to select for viable spores. Samples were then sonicated by microtip and diluted into CTT soft agar (0.5% agar) with the appropriate antibiotic treatment (40 μg/ml kanamycin, 5 μg/ml rifampin, 10 μg/ml novobiocin, or no antibiotic). In cases of mixed competitions where no colonies grew at our lowest dilution factor (10^1^), a value of ten spores/assay was entered for data analysis, providing conservatively low estimates of fitness inferiority in these cases.

In each competition mixture, the paired strains had distinct antibiotic-resistance marker states, allowing quantification of each competitor's spore production. In most cases, both competitors bore resistance to distinct antibiotics, allowing for direct mixing of one resistant representative from each strain. For two pairings (AB and GI), however, only variants with the same resistance marker were obtained. In these two cases, the resistant variants of both strains were mixed with the original antibiotic-sensitive clone of the respective competitor and their sporulation efficiencies in mixture were measured independently.

In the complete competition matrix, each of all possible pairwise competitions were performed in at least three temporally independent blocks, with two replicates of each particular competition performed within each block. Assays of pure-culture sporulation efficiency for all competitors were performed simultaneously with the respective competition assays, also with 2-fold replication within each block. Because variation across blocks was larger than within blocks, we conservatively used within-block means of relevant values as data points for statistical analysis.

The untransformed sporulation efficiency *(D)* of strain *i* in pure culture is the frequency of cells surviving development as viable spores:



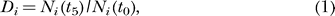



where *N_i_* represents the viable population size of *i* before starvation (*t*_0_) and after 5 d of starvation (*t*_5_). The efficiency of *i* in mixed competition with strain *j* is similarly given as



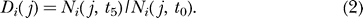



The effect of mixing strains *i* and *j* on the sporulation efficiency of strain *i* is given as



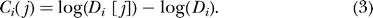



Thus, a positive value of *C_i_*(*j*) indicates that strain *i* sporulates more efficiently in the presence of strain *j* than in clonal isolation, whereas a negative value indicates that mixing with *j* negatively affects the efficiency of *i*. The relative mixing effect of strains *i* and *j* is given as



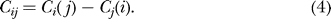



The predicted relative sporulation efficiency (log-transformed) of strains *i* and*j* during development assumes that there are no effects of mixing on sporulation efficiency and is defined as



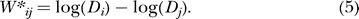



The actual relative sporulation efficiency (log-transformed) of two strains is defined as the log difference of the strains' actual sporulation efficiencies in mixed competition with each other:



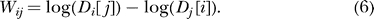



The bidirectional effect of mixing on total spore production *(B_ij_)* was calculated for all 36 matrix pairs as the frequency of spores produced in each mix *ij* relative to that expected from pure-culture assays of *i* and *j,* with



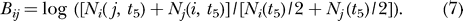



To estimate the significance of the negative overall mean of *B_ij_,* the average value of *B_ij_* for all eight pairs containing strain *i* was calculated for each of the nine strains. These nine means were used as statistical units in a one-sample *t*-test of the difference between the grand mean and the null expectation of zero, giving 8 *df*. Similarly, the mean *C_i_*(*j*) value of each strain (across each strain's eight pairings) was used as the statistical unit to estimate the significance of the overall one-way effect of mixing.

## Supporting Information

### Accession Number

The NCBI Entrez (http://www.ncbi.nlm.nih.gov/gquery/gquery.fcgi) accession number for DK1622 is M34114.
